# Commentary: Association of composite dietary antioxidant index with high risk of prostate cancer in middle-aged and elderly men: insights from NHANES

**DOI:** 10.3389/fimmu.2026.1799402

**Published:** 2026-03-02

**Authors:** Yu Dai, Shuangning Liu, Cheng Wang, Helin Zhang, Panfeng Shang

**Affiliations:** 1 Department of Urology, Baoji People’s Hospital, Baoji, Shaanxi, China; 2 Department of Urology, Lanzhou University Second Hospital, Lanzhou, Gansu, China

**Keywords:** benign prostatic hyperplasia (BPH), composite dietary antioxidant index(CDAI), diagnostic misclassification, NHANES, prostate cancer, prostate-specific antigen (PSA)

## Introduction

We read the article by Jin et al. (1) regarding the Composite Dietary Antioxidant Index (CDAI) and “high-risk prostate cancer” with great interest. While utilizing NHANES data offers valuable insights into nutritional epidemiology, we write to discuss potential methodological limitations that may affect the clinical applicability of their conclusions. Specifically, defining “high-risk prostate cancer” solely based on a serum tPSA > 10 ng/mL threshold in a cancer-free screening population raises concerns regarding oncological specificity.

## Distinction between clinical diagnosis and epidemiological surrogates

A primary concern lies in the definition of the outcome. In clinical practice, standard risk stratification requires histopathological confirmation (e.g., Gleason grading) and clinical staging (2). We acknowledge that in population-based research, biomarkers are often used as surrogate endpoints when pathological data is unavailable. However, even within an epidemiological framework, using a PSA threshold of >10 ng/mL as a sole proxy for “high-risk cancer” in older men is problematic. The 2023 AUA/SUO Guideline notes that PSA is a continuous variable that increases with both age and benign prostatic hyperplasia (BPH) (3). The EAU Guidelines also state that PSA’s continuous nature limits the utility of a single threshold for cancer detection (4). Consequently, without exclusion of benign causes, this surrogate endpoint likely captures a mixed signal of both malignancy and benign prostatic growth, potentially reducing the specificity of the findings.

## The anti-inflammatory hypothesis

The observed association between CDAI and PSA levels may also be interpreted through an alternative mechanism: the anti-inflammatory effect. Biological evidence suggests that inflammation modulates PSA levels in benign conditions. Cross-sectional data from Weng et al. (2023) indicate that pro-inflammatory diets are associated with PSA elevation (5). Furthermore, interventional studies, such as Schwarz et al. (2008), have observed that lycopene supplementation reduced serum PSA in patients with BPH (6). Similarly, Kutwin et al. (2022) highlighted how natural antioxidants may mitigate the oxidative stress driving BPH (7). We hypothesize that the inverse association reported by Jin et al. might plausibly reflect the efficacy of antioxidants in suppressing prostatic inflammation and lowering BPH-associated PSA, rather than exclusively indicating a protective effect against tumorigenesis.

## Epidemiological context and confounding factors

Finally, the prevalence of benign conditions in this demographic warrants consideration. The authors noted in their Introduction that PSA elevation is not specific to malignancy (1). Epidemiological data from the GBD 2019 study indicate that the prevalence of BPH in men aged 60 and older is substantial, often exceeding 50% (8), whereas the prevalence of prostate cancer is comparatively lower (<5%) in the general population. Given this prevalence gap, it is plausible that the “High-Risk” group (PSA > 10 ng/mL) includes a significant proportion of individuals with undiagnosed BPH. If BPH status was not fully adjusted for (potentially due to data limitations in NHANES), the study essentially compares a high-antioxidant group against a group with elevated PSA of mixed etiology.

## Discussion

In conclusion, while the CDAI is a valuable construct, the findings regarding “cancer prevention” should be interpreted with caution due to the non-specific nature of the PSA endpoint. We suggest that the results could be re-interpreted as an association between antioxidants and benign prostatic health or general PSA reduction. Future research utilizing NHANES data for oncological outcomes would benefit from integrating available diagnostic codes or specifying the limitations of biochemical surrogates to avoid potential misclassification.
